# Decreased miR-4512 Levels in Monocytes and Macrophages of Individuals With Systemic Lupus Erythematosus Contribute to Innate Immune Activation and Neutrsophil NETosis by Targeting TLR4 and CXCL2

**DOI:** 10.3389/fimmu.2021.756825

**Published:** 2021-10-14

**Authors:** Binbin Yang, Xinwei Huang, Shuangyan Xu, Li Li, Wei Wu, Yunjia Dai, Ming-Xia Ge, Limei Yuan, Wenting Cao, Meng Yang, Yongzhuo Wu, Danqi Deng

**Affiliations:** ^1^ Department of Dermatology, The Second Affiliated Hospital of Kunming Medical University, Kunming, China; ^2^ Key Laboratory of The Second Affiliated Hospital of Kuming Medical University, Kunming, China; ^3^ Department of Dermatology, The 6th Affiliated Hospital of Kunming Medical University, The People’s Hospital of Yuxi City, Kunming, China; ^4^ Dai Medicine College, West Yunnan University of Applied Sciences, Xishuangbanna, China; ^5^ Department of Dermatology, Suining Central Hospital, Suining, China; ^6^ Department of Dermatology, Panlong District People’s Hospital, Kunming, China; ^7^ State Key Laboratory of Genetic Resources and Evolution/Key Laboratory of Healthy Aging Research of Yunnan Province, Kunming Institute of Zoology, Chinese Academy of Sciences, Kunming, China; ^8^ Kunming College of Life Science, University of Chinese Academy of Sciences, Beijing, China

**Keywords:** systemic lupus erythematosus, miR-4512, CXCl2, neutrophil extracellular trap, innate immune activation

## Abstract

**Objective:**

Systemic lupus erythematosus (SLE) is an autoimmune disease with complex etiology that is not yet entirely understood. We aimed to elucidate the mechanisms and therapeutic potential of microRNAs (miRNAs) in SLE in a Tibetan population.

**Methods:**

Peripheral blood mononuclear cells from SLE patients (n = 5) and healthy controls (n = 5) were used for miRNA–mRNA co-sequencing to detect miRNAs related to immune abnormalities associated with SLE. Luciferase reporter assay was used to identify potential targets of candidate miRNA. The target genes were verified in miRNA-agomir/antagomir transfection assays with multiple cells lines and by expression analysis. The effects of candidate miRNA on monocyte and macrophage activation were evaluated by multiple cytokine profiling. Neutrophil extracellular traps (NETs) formation was analyzed *in vitro* by cell stimulation with supernatants of monocytes and macrophages transfected with candidate miRNA. The rodent MRL/lpr lupus model was used to evaluate the therapeutic effect of CXCL2Ab on SLE and the regulation effect of immune disorders.

**Results:**

Integrated miRNA and mRNA expression profiling identified miRNA-4512 as a candidate miRNA involved in the regulation of neutrophil activation and chemokine-related pathways. MiR-4512 expression was significantly reduced in monocytes and macrophages from SLE patients. MiR-4512 suppressed the TLR4 pathway by targeting *TLR4* and *CXCL2*. Decreased monocyte and macrophage miR-4512 levels led to the expression of multiple proinflammatory cytokines *in vitro*. Supernatants of miR-4512 antagomir-transfected monocytes and macrophages significantly promoted NETs formation (*P* < 0.05). Blocking of CXCL2 alleviated various pathogenic manifestations in MRL/lpr mice, including kidney damage and expression of immunological markers of SLE.

**Conclusions:**

We here demonstrated the role of miR-4512 in innate immunity regulation in SLE. The effect of miR-4512 involves the regulation of monocytes, macrophages, and NETs formation by direct targeting of *TLR4* and *CXCL2*, indicating the miR-4512-TLR4-CXCL2 axis as a potential novel therapeutic target in SLE.

## Introduction

Systemic lupus erythematosus (SLE) is a chronic, incurable autoimmune disease. It is characterized by the induction of abnormal cell death pathways and clearance mechanisms, excessive externalization of modified cells and nuclear debris, loss of tolerance to various self-antigens, and innate and adaptive immune disorders (1). The innate immune system might initiate and promote SLE autoimmunity and organ damage (2). Pro-inflammatory monocytes, macrophages, and neutrophils enter the kidney and other organs during SLE progression (3). Further, abnormal macrophage activation and secretion have been demonstrated in SLE in human and in animal models (4). However, the mechanism by which the innate immune system, especially pro-inflammatory monocytes, macrophages and neutrophils cause organ damage in SLE is not yet fully understood.

MicroRNAs (miRNAs) are small non-coding RNAs that act by regulating gene expression. The roles of miRNAs in regulating immune defense genes remain to be clarified (5). Some information on miRNA activity in SLE is available. For instance, miRNA-30e-5p regulates the innate immune response during viral infection and SLE (6). MiR-146a suppresses nuclear factor-k-gene binding (NF-κB) and cytokine production during SLE development (7). MiR-155 targets the suppressor of cytokine signaling 1 (SOCS1) in macrophages and promotes an inflammatory response and type I interferon (IFN) signaling (8). MiRNA-150 promotes inflammation by suppressing the triggering receptors expressed on myeloid cells 1 (TREM-1), which are positive regulators of the toll-like receptor 4 (TLR-4) signaling pathway (9). MiR-125a regulates autoimmune diseases by stabilizing immune homeostasis mediated by regulatory T cells (10). However, the roles of miRNAs in monocytes and macrophages during autoimmune pathogenesis remain to be elucidated.

We have previously shown that the expression of miR-125b-5p decreases and that of the autophagy gene UVRAG increases, activating cell autophagy after UVB irradiation of the peripheral blood mononuclear cells (PBMCs) of SLE patients of Han ethnicity (11). Tibetans are theoretically more susceptible to systemic lupus than Han Chinese because they live at high altitudes and are exposed to oxygen deprivation and intense ultraviolet radiation. However, the characteristics of gene expression in Tibetan patients with SLE in China have not been reported. In the current study, blood samples from Tibetan patients with SLE in Diqing Shangri-La (Yunnan Province, China) were analyzed by high-throughput sequencing to screen for mRNA and miRNAs differentially expressed between healthy and SLE Tibetan patients. Next, miRNA-mRNA co-sequencing and functional analysis revealed that miRNA-4512 may be involved in immune regulation of SLE. We analyzed the expression of miR-4512 in PBMCs and monocytes of Tibetan and Han SLE patients, and identified the mechanism and direct target of miR-4512 in innate immune regulation. In addition, the therapeutic efficacy of these targets in animal models of SLE was further verified.

## Materials and Methods

### Patients and Controls

Nineteen SLE patients (10 Tibetan, mean age 46.1 ± 16.42 y; nine Han, mean age 35 ± 13.67 y) and 17 healthy controls (nine Tibetan, mean age 39.70 ± 3.76 y; eight Han, mean age 29.78 ± 1.37 y) matched for age, gender, and altitude were recruited (**Supplementary Table S1**). All patients met ≥ 4 of the revised SLE criteria of the American College of Rheumatology (1997) (12). Informed written consent was obtained, and the study design was approved by the Ethics Committee of the Second Affiliated Hospital of Kunming Medical University.

### RNA Extraction and Reverse-Transcription Quantitative Polymerase Chain Reaction (RT-qPCR)

Total RNA from freshly isolated PBMCs (section 2.7) or cell cultures was extracted with TRIzol Reagent (Invitrogen, San Diego, CA, USA). The cDNA was synthesized using a SureScript™ First-Strand cDNA Synthesis Kit (GeneCopoeia, Guangzhou, China). The mRNA levels were determined by RT-qPCR with BlazeTaq™ SYBR^®^ Green qPCR Mix 2.0 (GeneCopoeia) and *GAPDH* as the internal control. The primers are listed in **Supplementary Table S2**. Total RNA was reverse-transcribed and quantified with an All-in-One™ miRNA RT-qPCR Detection kit (QP015, GeneCopoeia). U6 small nuclear RNA was the internal control.

### Sample Library Construction and Sequencing

Total RNA was extracted from samples before sequencing using TRIzol reagent (Invitrogen, San Diego, CA, USA). RNA integrity and quantity were evaluated by using a Nanodrop spectrophotometer (Thermo Fisher Scientific, Waltham, MA, USA) and Agilent 2100 Bioanalyzer (Agilent Technologies, Palo Alto, California, USA). The cDNA was synthesized using Superscript II reverse transcriptase (Invitrogen, Carlsbad, CA, USA) and random primers, with enriched and fragmented RNA as template. The cDNA was converted to dsDNA for library construction. The libraries were quantified using an Agilent 2100 Bioanalyzer (Agilent Technologies, Palo Alto, California, USA). Sequencing was performed using Illumina NextSeq6000 platform (Illumina, San Diego, CA, USA). Multiplex Small RNA Library Prep Set for Illumina was used to construct small RNA libraries based on miRNA sequencing, in accordance with the manufacturer’s protocol. Fragments (18-35 nt) were extracted from the total RNA, and indices and adaptors were added for PCR amplification. Agilent 2100 Bioanalyzer (Agilent Technologies, Palo Alto, CA, USA) was used for library quantification. The library was sequenced by Shanghai Personal Biotechnology Co. Ltd. (Shanghai, China) using Hiseq platform (Illumina).

### Sequencing Data Analysis

The reference genome index was set up using Bowtie2 v. 2.4.1 (http://bowtie-bio.sourceforge.net/bowtie2/index.shtml). HISAT2 v. 2.1.0 (http://www.ccb.jhu.edu/software.shtml) was used to map the filtered reads to the reference genome. Reads for each gene were counted using HTSeq v. 0.11.1 (https://htseq.readthedocs.io/en/master/). Gene expression was standardized using FPKM, and differential expression was analyzed using DESeq v. 1.39.0 (http://bioconductor.org/packages/release/bioc/html/DESeq.html) according to the criteria |log2FoldChange| > 1 and *P* < 0.05. MiRDeep2 v. 2.0.0.8 (https://github.com/Rajewsky-lab/mirdeep2.git) was used to map the clean reads to the reference genome in the miRBase database (http://www.mirbase.org/), to annotate unique reads. Other non-coding RNAs were used for the annotation. Mireap v. 2.0 (https://sourceforge.net/projects/mireap/) was used to analyze the unannotated sequences. MiRNA reads were counted based on the number of sequences conforming with mature miRNA. The most abundant miRNAs were selected for subsequent analysis. DESeq v. 1.30.0 was used to identify differentially expressed miRNAs as those meeting the criteria |log2FoldChange| > 1 and *P* < 0.05.

The mRNA 3′ untranslated region (UTR) was the target sequence. MiRanda v. 3.3a (https://anaconda.org/bioconda/miranda) was used to predict the target genes of the differentially expressed miRNAs. Kyoto Encyclopedia of Genes and Genomes (KEGG, http://www.kegg.jp/) and gene ontology (GO, http://geneontology.org/) were used to analyze the enrichment of target genes associated with differentially expressed mRNA and miRNAs. All genes were mapped to KEGG and GO terms and pathways, and the latter were filtered using corrected *P* ≤ 0.05.

### Luciferase Reporter Assay

To verify the target genes of miR-4512, putative miR-4512 target sequences located in the 3′-UTR of human C-X-C motif ligand (*CXCL2*, *TLR4*, *CCR1*, *CXCL1*, and *CXCL5* transcripts were synthesized and cloned into pmirGLO vector (Promega, Madison, WI, USA) downstream of the *Luc2* reporter gene. Plasmids harboring mutated sequences were constructed by inserting each target sequence with various base deletions at the putative miR-4512–binding site (The sequences are listed in **Supplementary Figure S1**). Vectors were sequenced and prepared using an Endo-Free Plasmid ezFlow Mini kit (Promega). Then, HEK293T cells were co-transfected with pmirGLO-(CXCL2, TLR4, CCR1, CXCL1 and CXCL5) and pmirGLO-(CXCL2, TLR4, CCR1, CXCL1 and CXCL5)-MUT plasmids using Lipofectamine™ 2000 (Invitrogen, Carlsbad, CA, USA) and miR-4512 agomir or miR-4512 NC. After 48 h, the cells were harvested, and relative luciferase activity was measured using a Dual-Luciferase Reporter Assay System (Promega, Madison WI, USA), according to the manufacturer’s instructions. Renilla luciferase normalized firefly luciferase activity.

### Enzyme-Linked Immunosorbent Assay (ELISA), Western Blotting

The levels of various cytokines [interleukin (IL)-1β, IL-2, IL-12, tumor necrosis factor (TNF)-α, IFN-γ, IL-4, IL-6, IL-10, and IL-17] in the sera of human patients and experimental mice, supernatants of monocytes or macrophages transfected with NC/miR-4512-agomir/antagomir and mouse renal lysis solution were quantified using ELISA kits (NeoBioscience, Beijing, China), according to the manufacturer’s instructions. Mouse serum antinuclear antibody (ANA), anti-dsDNA antibody, and human serum CXCL2 concentrations were measured using appropriate ELISA kits (JL17316-48T, JL12477-48T, and JL25367-96T, respectively; Jianglai Biology, Shanghai, China). Human serum myeloperoxidase (MPO) concentration was measured with an ELISA kit (0-111594; Jonln, Shanghai, China), according to the manufacturer’s instructions. Anti-TLR4 monoclonal antibody (1:1000, clone 3G9A4; Proteintech Group, Chicago, IL USA) and Anti-neutrophil elastase (NE) monoclonal antibodies (1:100; 89241S; Cell Signaling Technology, Danvers, MA, USA) were used for western blotting, with β-actin was the internal control.

### Cell-Free DNA (cfDNA) Detection

Cell supernatant was treated according to the Picogreen Kit (Invitrogen), and each sample was diluted 1:9 (cell supernatant: TE working solution) with TE working solution; Add 100ul of 10-fold diluted samples to each well, set up two duplicate Wells, and then add 200ul Picogreen reagent in an aqueous working solution chamber to incubate at warm temperature for 10min; Determination: The full-function continuous wavelength scanning microplate analysis system measured all samples in a 96-well microplate at a final volume of 200ul. The standard curve was established with the concentration of DNA standard as the X axis and the fluorescence at 480nm as the Y axis. The concentration of the corresponding sample was calculated according to the fluorescence value of the sample.

### Cell Isolation and Culture

PBMCs were separated from ethylene diamine tetraacetic acid (EDTA) blood by density gradient centrifugation (450 × *g*, 30 min) on Ficoll-Paque Plus (GE Healthcare, Little Chalfont, UK) and resuspended in RPMI 1640 medium supplemented with 100 U/mL penicillin and 100 μg/mL streptomycin (Gibco, Grand Island, NY, USA) and 10% fetal bovine serum (Gibco, Grand Island, NY, USA).

CD14^+^CD16^–^ monocytes were isolated from freshly isolated PBMCs by immunomagnetic negative selection using an EasySep™ Human Monocyte Isolation kit (19669; STEMCELL Technologies, Vancouver, BC, Canada), according to the manufacturer’s instructions. Human macrophages were obtained by culturing CD14^+^ monocytes for 7 d in RPMI 1640 medium containing 50 ng/mL macrophage colony-stimulating factor(M-CSF). The macrophages were identified by F4/80 expression. Neutrophils were isolated by gradient centrifugation (500 × *g*, 30 min) on Hypaque-Ficoll solution (LZS11131; TBD, Tianjin, China). Monocytes, neutrophils, and differentiated macrophages were cultured in complete RPMI 1640 medium.

### *In Vitro* NETs Formation Assay

CD14^+^ monocytes from healthy donors and macrophages were transfected with NC/miR-4512-agomir/antagomir using EndoFectin™ Max (EF013; Genecopoeia). After 12 h, the conditioned medium was collected. Fresh neutrophils isolated from healthy donors were exposed to the medium for 3 h. Cells treated with 10 μg/mL lipopolysaccharide (LPS) and 50 ng/mL CXCL2 were used as the positive controls. Culture supernatants were collected for ELISA analysis. Stimulated neutrophils were collected for western blotting and NETs staining and quantification.

### NETs Staining and Quantification

The neutrophils were inoculated in the confocal dishes at a concentration of 2×10^5^/mL, fixed with 4% paraformaldehyde for 15 min, then permeabilized with 0.5% Triton X-100 at room temperature for 10 min. Then the cells were stained with 1 μm Sytox Green (Invitrogen) and Hoechst nucleic acid stain (1:1000; Invitrogen). Random photographs were taken under the condition of scattered light at 520 nm. The obtained images were analyzed by Zen 2012 software (n=4). For quantification of NETs, neutrophils were seeded in 96-well black plates (2 × 10^5^ cells/well) in the presence of 5 μM Sytox Green. After stimulation with different conditioned medium for 3 hours, the florescence intensity was quantified with excitation at 485 nm and emission at 535 nm using Infinite M200 Pro (Tecan).

### MLR/lpr Animal Model

Four female MRL/lpr mice (8-wk-old, 19 ± 0.5 g; Hunan Slack Jingda Experimental Animal Co. Ltd., Hunan, China) were intraperitoneally injected with mouse CXCL2 monoclonal antibody (300-39-2; PeproTech, Rocky Hill, NJ, USA), twice weekly (50 μg/50 μL) for 4 wk. The control (n = 4, female MRL/lpr mice, 8-wk-old, 18 ± 0.5 g; Hunan Slack Jingda Experimental Animal Co. Ltd.) received 50 μL of saline per treatment. Sex- and age-matched normal C57BL/6 mice (n = 4, 8-wk-old, 18 ± 0.5 g; Hunan Slack Jingda Experimental Animal Co. Ltd.) were administered saline according to the above schema. Urine was collected twice weekly from each group. Proteinuria was detected by Coomassie Brilliant Blue (CBB) staining (Bayer, Elkhart, IN, USA). Mice were sacrificed by CO_2_ asphyxiation at 12 wk. The blood was collected, and serum levels of anti-dsDNA, ANA and multiple cytokines were measured using commercial kits (Jianglaibio, Shanghai, China). The kidneys were excised and dissected for immunocytochemistry (IHC) staining (as described in section 2.10) and cytokine analysis by ELISA.

The mice were randomly assigned to different groups according to their unique code, and all data were collected by technicians blinded to the treatment protocol. The experiment was conducted in a laboratory animal facility of Kunming Medical University and approved by the Animal Research Committee of Kunming Medical University (No. kmmu2021724).

### Histopathology and IHC Staining

Mouse kidneys were fixed in 4% (v/v) paraformaldehyde, embedded in paraffin, sectioned (6 μm) using a rotary microtome (Leica Biosystems, Wetzlar, Germany), and stained with hematoxylin and eosin (H&E). Active and chronic renal tubular changes were semi-quantitatively analyzed as previously described (13). The activity index (AI) data included glomerular endocapillary proliferation, intraglomerular leukocyte infiltration, platinum ear-pick and/or hyaline thrombus, fibrinoid necrosis and/or nuclear fragmentation, cellular crescent, and inflammatory cell infiltration in the interstitial space. A score in the range of 0–3 was assigned to each item, and all items were summed to yield a final AI score. The chronic index (CI) parameters included glomerulosclerosis, fibrous crescent, renal tubular atrophy, and renal interstitial fibrosis. A score in the range of 0-3 was assigned to each item, and all items were summed to yield a final CI score. Four items in the renal tubulointerstitial lesion (TIL) category were evaluated: tubular degeneration or necrosis, renal tubular atrophy, renal interstitial fibrous, and renal interstitial inflammatory cell infiltration. A score in the range of 0–3 was assigned to each item, and all items were summed to yield a final TIL score. Tissue sections were examined by an experienced pathologist blinded to the treatments.

For IHC staining, renal paraffin sections were dewaxed, rehydrated, and recovered in antigen recovery solution (ETDA with citric acid) in an autoclave. Endogenous peroxidase activity was blocked with 0.3% (v/v) hydrogen peroxide. The sections were blocked with 10% (v/v) normal goat serum at 37°C for 1 h and incubated with rabbit anti-CXCL2 monoclonal antibody (MAb) (1:200; GTX74085; GeneTex, Irvine, CA, USA), rabbit anti-CD45 MAb (1:1000; 20103-1-AP; Proteintech, Chicago, IL, USA), and rabbit anti-MPO MAb (1:1000; ab188211; Abcam, Cambridge, UK) at 4°C overnight. Tissues were washed with phosphate-buffered saline, incubated with horseradish peroxidase-conjugated secondary antibody, and analyzed by using a DAB kit. The nuclei were counterstained with hematoxylin. Whole-tissue images were acquired using a Pannoramic 250 flash II scanner (3D HISTECH, Budapest, Hungary) at ×20magnification. According to the intensity of immunohistochemical staining, the glomeruli were divided into four grades. Specifically, deposition <20%, 20% ≤ deposition <50%, 50% ≤ deposition <75%, and deposition ≥ 75 are defined as degrees I, II, III, and IV, respectively. Data are expressed as the percentage of glomeruli in each staining category relative to the total. Three sequential sections per animal were examined, and averages were statistically analyzed.

### Statistical Analysis

Data are presented as the mean ± SEM. For normal variance distribution, unpaired Student’s *t*-test was used to determine the differences between group pairs. One-way ANOVA and *post hoc* Kruskal–Wallis test were used to compare ≥ 3 groups. GraphPad Prism 5 (GraphPad Software, La Jolla, CA, USA) was used for the calculations. Statistical significance was set at *P* < 0.05.

## Results

### Integrated miRNA and mRNA Expression Profiling Identifies Putative miRNA-mRNA Regulatory Networks in PBMCs From SLE Patients

**Figure 1:**
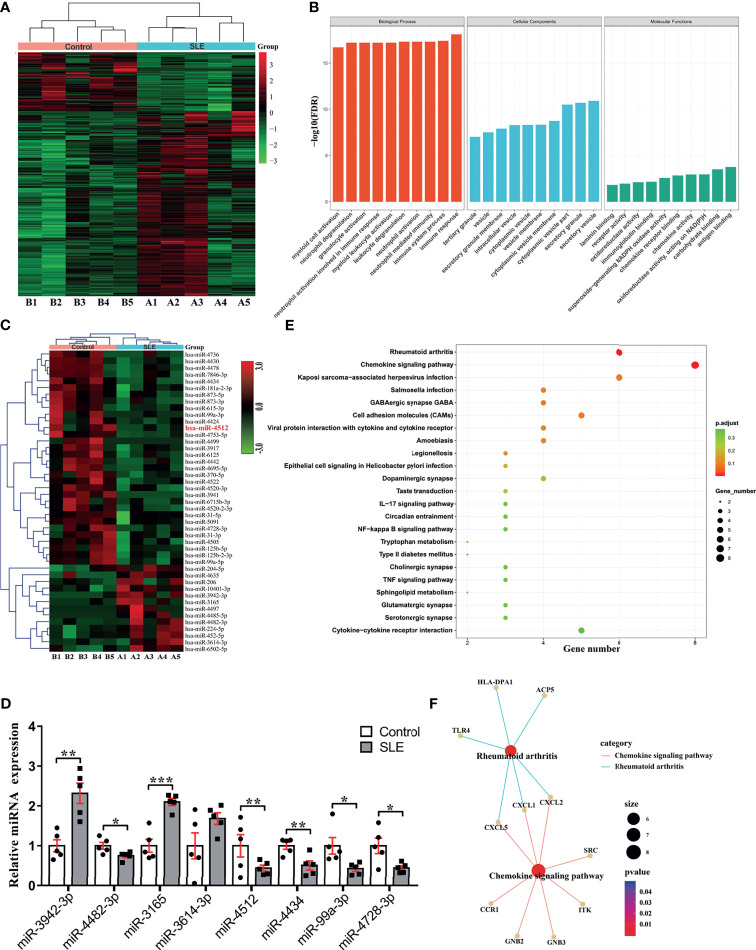
miRNA and mRNA profiling reveals potential regulatory network involved in SLE pathogenesis. **(A)** Heat map representation of differentially expressed genes in SLE patients and control subjects (n = 5). **(B)** Top 10 GO processes determined based on all differentially expressed genes highlight enrichment of neutrophil activation and chemokine activity. **(C)** Hierarchical clustering of 45 differentially expressed miRNAs in SLE patients and control subjects from the same cohort determined by mRNA sequencing (>2-fold change, *P* < 0.05). **(D)** The top four downregulated and upregulated miRNAs were analyzed by RT-qPCR in PBMCs from five SLE patients and five age- and gender-matched healthy controls. PBMC set different from that used in **(C)** was analyzed. Results are presented as the mean ± SEM. ***P < 0.001; **P < 0.01; *P < 0.05. **(E)** KEGG analysis of predicted target genes of miR-4512. **(F)** The regulatory networks of miR-4512 target genes and autoimmune disease-related genes.

We used RNA sequencing to analyze miRNA and mRNA expression profiles of PBMCs from SLE (n = 5) and healthy (n = 5) Tibetans. Overall, 469 mRNAs (DEmRNAs) and 45 miRNAs (DEmiRNAs) were significantly differentially expressed in Tibetan SLE patients. Of these, 356 DEmRNAs and 13 DEmiRNAs were upregulated, while 113 DEmRNAs and 32 DEmiRNAs were downregulated (**Figures 1A, C**).

To identify the functional differences between SLE and healthy patients, we used the 469 DEmRNAs in GO enrichment analysis. We thus obtained top 10 GO terms related to Biological Processes (BP), Molecular Functions (MF), and Cellular Components (CC) at *P* < 0.05 (**Figure 1B**). Most items in the BP category were related to immune and neutrophil activation and degranulation. Chemokine activity and multiple cellular secretion-related components were enriched in the MF and CC categories, respectively. Hence, GO enrichment analysis suggested that neutrophil migration and activation participate in SLE pathogenesis.

To elucidate the regulatory mechanism of DEmRNAs in SLE, we investigated their relationships with DEmiRNAs. We used RT-qPCR to validate four downregulated and four upregulated DEmiRNAs with the largest expression fold changes in PBMCs isolated from five Tibetan SLE patients, and five age- and sex-matched Tibetan controls. MiR-3942-3p, miR-3165 and miR-3614-3p were upregulated, while miR-4482-3p, miR-99a-3p, miR-4728-3p, miR-4512 and miR-4434 were downregulated in PBMCs from Tibetan SLE patients (**Figure 1D**). We predicted the target genes of these candidate miRNAs using TargetScan (http://www.targetscan.org). The analysis revealed 332 target genes of 469 DEmRNAs, miR-4512 was predicted to target the largest number of genes.

According to the KEGG analysis, the DEmRNAs targeted by hsa-miR-4512 were significantly enriched in the chemokine signaling, rheumatoid arthritis-associated, NF-kB signaling, and other immune inflammatory pathways (*P* < 0.05) (**Figure 1E**), while the DEmRNAs targeted by other candidate miRNAs were not enriched in any immune-related pathways (**Supplementary Figure S2**). *CCR1*, *CXCL1*, *CXCL2*, *CXCL5*, and *TLR4* mRNA were negatively correlated with miR-4512 in the immune inflammatory pathways, and we used them to construct regulatory networks (**Figure 1F**). Thus, hsa-miR-4512 might regulate neutrophil activation and chemokine-related pathways.

### miR-4512 Inhibited The TLR4 Pathway by Targeting TLR4 and CXCL2

**Figure 2:**
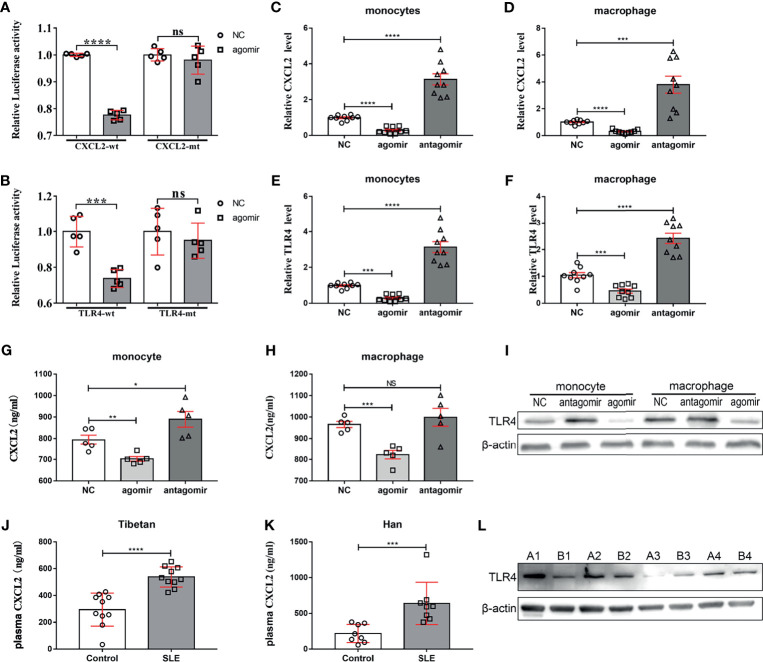
miR-4512 directly targets *TLR4* and *CXCL2*. **(A, B)** Luciferase activity of WT/MUT-CXCL2 and WT/MUT-TLR4 plasmids in HEK293T following transfection with an miR-4512 agomir. Data are presented as the mean ± SEM. ****P < 0.0001; ***P < 0.001; NS = not significant. **(C–F)** RT-qPCR analysis of *CXCL2* and *TLR4* transcript levels in human primary monocytes and macrophages 48 h after transfection with NC/miR-4512 agomiR/antagomiR. Data are presented as the mean ± SEM. ****P < 0.0001; ***P < 0.001. **(G, H)** CXCL2 concentration in the supernatants obtained from human primary monocytes **(G)** and macrophages **(H)** 48 h after transfection with NC/miR-4512-agomir/antagomir. Data are presented as the mean ± SEM. ***P < 0.001; **P < 0.01; *P < 0.05; NS, not significant. **(I)** Western blot analysis of TLR4 expression in monocytes and macrophages 72 h after transfection with NC/miR-4512-agomir/antagomir. Results are representative of three independent experiments, with actin used as an internal control. **(J, K)** CXCL2 concentration in the plasma of SLE and control subjects from Tibetan **(J)** and Han **(K)** populations. Data are presented as the mean ± SEM. ****P < 0.0001; ***P < 0.001. **(L)** Western blot analysis of TLR4 expression in PBMCs isolated from SLE patients (A1–A4) and healthy controls (B1–B4). Results are representative of three independent experiments.

TargetScan and KEGG pathway analysis were used to narrow the range of miR-4512 predicted targets based on differentially expressed mRNAs associated with miR-4512 in immune-inflammatory pathways. The recognized target genes of miR-4512 were used in pathway analysis based on CapitalBio molecular annotation system v.3.0. The prediction results suggested that CCR1, CXCL1, CXCL2, CXCL5, and TLR4 might be the target of miR-4512 (**Figure 1F**). Luciferase reporter plasmid containing mRNA sequences of WT or mutated 3’-UTR target genes was co-transfected into HEK293T cells with NC and miR-4512 agomir. Only TLR4 and CXCL2 were directly targeted by miR-4512 (**Figures 2A, B**). RT-qPCR analysis showed that TLR4 and CXCL2 were also negatively regulated by miR-4512 in BALL-1, Jurkat and K562 cells, as well as in human primary monocytes and macrophages (**Figures 2C–F** and **Supplementary Figure S3**). In addition, miR-4512 inhibited the expression of CXCL2 and TLR4 proteins in human primary monocytes and macrophages (**Figures 2G–I**). The elevated serum CXCL2 level and the high expressed TLR4 in PBMCs of another group of SLE patients indicate that the down-regulation of miR-4512 in SLE promotes the expression of CXCL2 and TLR4 (**Figures 2J–L**).

### miR-4512 Downregulation in Monocytes and Macrophages Contributes to The Pro-Inflammatory Condition

**Figure 3:**
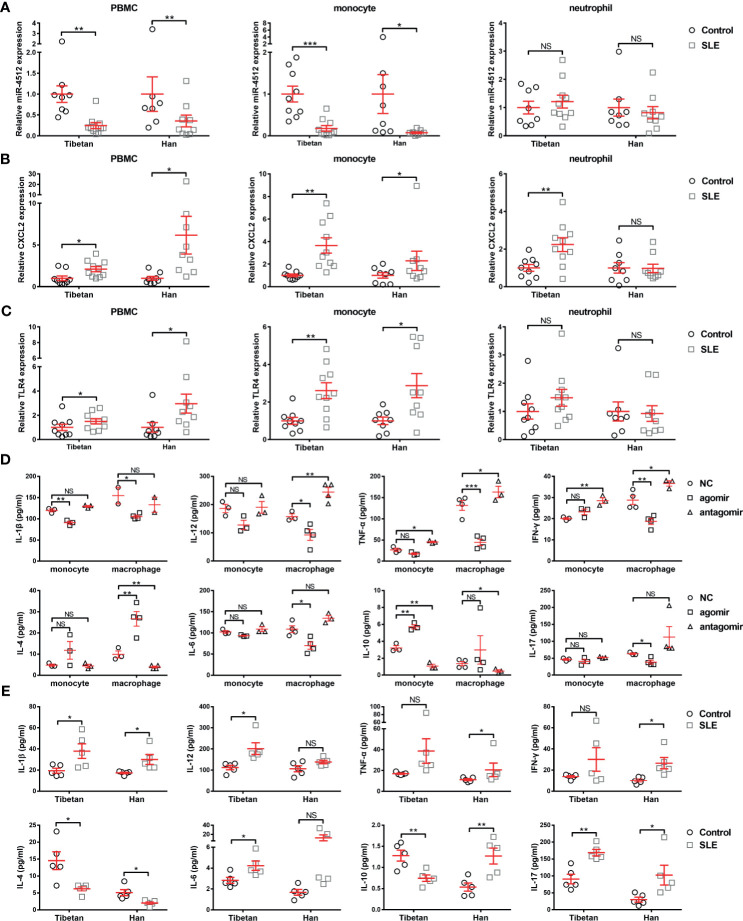
Decreased miR-4512 expression in monocytes and macrophages contributes to a pro-inflammatory state. **(A–C)** Relative expression of miR-4512 **(A)**, *CXCL2* **(B)**, and *TLR4* **(C)** in PBMC, monocytes, and neutrophils isolated from SLE patients and control subjects, as determined by RT-qPCR. Data are presented as the mean ± SEM. ***P < 0.001; **P < 0.01; *P < 0.05; NS, not significant. **(D–E)** Concentrations of Th1 (including IL-1β, IL-12, TNF-α, and IFN-γ), Th2 (including IL-4, IL-6, and IL-10), and Th17 (IL-17) cytokines in the supernatants obtained from monocytes and macrophages transfected with NC/miR-4512-agomir/antagomir **(D)** or in the serum from SLE patients and control subjects **(E)**, as determined by ELISA.

Considering the direct regulation of *TLR4* and *CXCL2* by miR-4512, one could speculate that miR-4512 mainly plays a role in cells which express sufficient TLR4, such as monocytes, granulocytes, and macrophages (14). Accordingly, we isolated PBMCs, monocytes, and neutrophils from Tibetan and Han SLE patients and their matched normal controls. RT-qPCR analysis of miR-4512, *CXCL2*, and *TLR4* levels revealed that miR-4512 levels were decreased in monocytes and macrophages, but not neutrophils, from SLE patients (**Figures 3A–C**).

We then analyzed the impact of miR-4512 downregulation on cytokine secretion in monocytes and macrophages. Primary monocytes and macrophages pooled from healthy subjects were transfected with NC/miR-4512-agomir/antagomir, and the cytokines in their cell culture supernatants were quantified. As shown in **Figure 3D**, miR-4512 inhibited the secretion of pro-inflammatory cytokines IL-1β, IL-12, TNF-α, IFN-γ, and IL-17 and promoted the secretion of anti-inflammatory cytokines IL-4 and IL-10. This finding was consistent with the cytokine secretion phenotype of SLE patients (**Figure 3E**). Therefore, miR-4512 downregulation in SLE patient monocytes and macrophages may activate monocytes and macrophages and induce pro-inflammatory cytokine secretion.

### MiR-4512-Transfected Monocytes and Macrophages Inhibit Neutrophil NETosis

**Figure 4:**
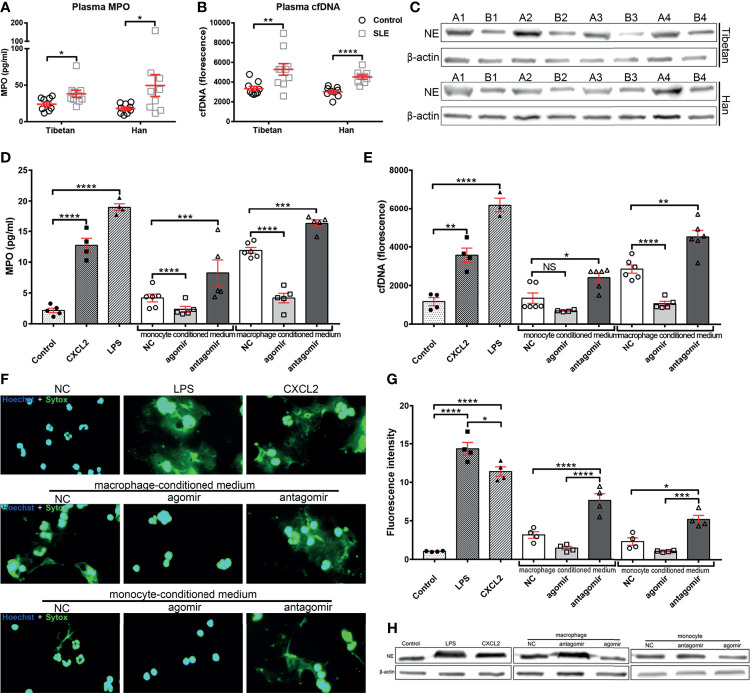
Supernatants of monocytes and macrophages transfected with miR-4512 promote neutrophil extracellular traps formation. **(A, B)** Plasma levels of myeloperoxidase (MPO) **(A)** and cell-free DNA (cfDNA) **(B)** in SLE patients and control subjects from Tibetan and Han populations. Data are presented as the mean ± SEM. ****P < 0.0001, **P < 0.01, *P < 0.05. **(C)** Western blot analysis of neutrophil elastase (NE) expression in neutrophils isolated from SLE patients (A1–A4) and control subjects (B1–B4) from Tibetan and Han populations. Results are representative of three independent experiments, with actin used as an internal control. **(D, E)** MPO **(D)** and cfDNA **(E)** levels in the supernatants of human primary neutrophils culture stimulated with a supernatant of monocytes and macrophages transfected with NC/miR-4512-agomir/antagomir. Neutrophils directly stimulated with LPS or CXCL2 were used as positive controls. Data are presented as the mean ± SEM. ****P < 0.0001; ***P < 0.001; **P < 0.01; *P < 0.05; NS, not significant. **(F)** Images of neutrophils stimulated with macrophage and monocyte conditioned medium or with CXCL2 and LPS. Nuclei were stained with Hoechst (blue) and the presence of extracellular DNA were stained with Sytox Green. Images are representatives of three independent experiments. **(G)** Quantification of neutrophils extracellular DNA during NETosis by Sytox Green assays. Data are presented as the mean ± SEM. ****P < 0.0001; ***P < 0.001; *P < 0.05. **(H)** Western blot analysis of elastase expression in neutrophils after stimulation. Images are representatives of three independent experiments.

Defective apoptotic debris clearance and NETs degradation might lead to the formation of autoantibodies in SLE pathogenesis (15). As miR-4512 downregulation in monocytes and macrophages promotes pro-inflammatory cytokine secretion and CXCL2 expression, miR-4512 may indirectly prime neutrophils for NETosis. As shown in **Figures 4A, B**, the levels of NETs-relevant indicators [MPO and cfDNA] were elevated in SLE patient sera. The levels of NE, the main NETs component, were significantly upregulated in neutrophils isolated from SLE patients (**Figure 4C**). Hence, dysregulation of NETs release and/or defective NETs clearance are involved in the pathogenesis of SLE.

NC/miR-4512-agomir/antagomir was then transfected into primary monocytes and macrophages to determine the role of miR-4512 on the formation of NETs. 12 h after transfection, the supernatants of each group were isolated and used to stimulate neutrophils isolated from the healthy control group. As shown in **Figures 4D, E**, the supernatants of monocytes and macrophages transfected with miR-4512-agomir significantly reduced MPO and cfDNA release by neutrophils. The effect of the supernatant from monocytes and macrophages transfected with miR-4512-antagomir on neutrophil NETosis reached a level comparable to that of the LPS stimulation group. Straining and quantification of extracellular DNA with SYTOX Green (**Figures 4F, G** **)** and Western-Blot analysis of NE in the neutrophils (**Figure 4H**) also indicated similar results. Remarkably, CXCL2-stimulation also promoted neutrophil NETosis, suggesting that miR-4512 present in monocytes and macrophages may modulate neutrophil NETosis through chemoattraction and activation of neutrophils by secreting CXCL2.

### CXCL2 Blocking Alleviates Kidney Damage in MRL/lpr Mouse

**Figure 5:**
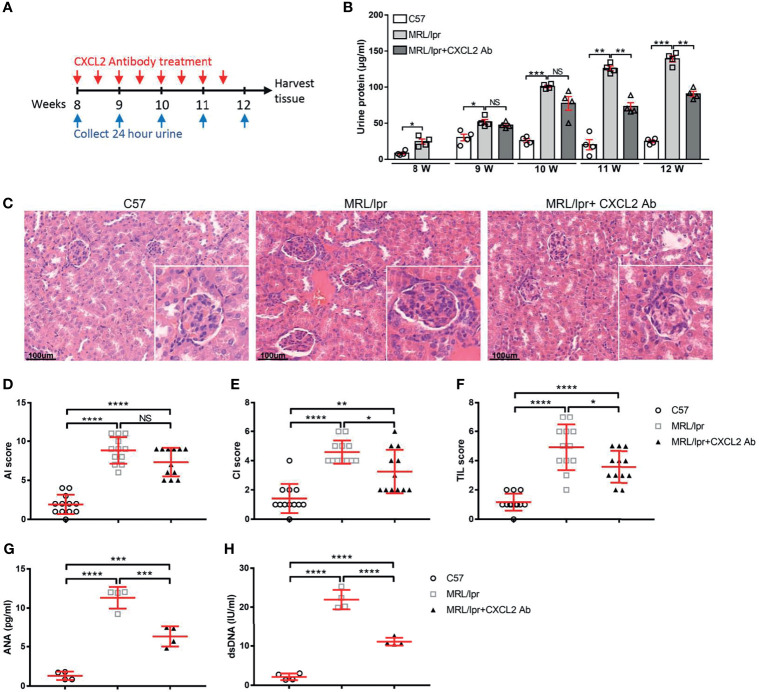
Blocking of CXCL2 alleviates kidney damage in MRL/lpr mice. **(A)** Treatment schema: red arrows indicate days of CXCL2 neutralizing antibody treatment, and the blue arrows indicate time points for detection of 24 urinary proteins. **(B)** Urine of mice from different groups was collected at the indicated time points for the proteinuria assay. ***P < 0.001; **P < 0.01; *P < 0.05; NS, not significant. **(C)** Representative images of H&E straining of kidney sections obtained from mice from different groups. Inserts, details of a typical glomerulus. **(D–F)** Lesion scores of the activity index **(D)**, chronicity index **(E)**, and tubulointerstitial lesions **(F)** of mice in different groups. Data are shown as the mean ± SEM (n = 4 animals/group, 3 sections/animal). ****P < 0.0001; ***P < 0.001; *P < 0.05. **(G, H)** Immunological markers of SLE in the serum of mice from different groups. The antinuclear antibodies (ANA) were determined by immunofluorescence **(G)**, anti-dsDNA levels were measured by ELISA **(H)**. ****P < 0.0001; ***P < 0.001; **P < 0.01.

In order to verify the function of miR-4512 in SLE pathogenesis, we blocked the target gene CXCL2 with a neutralizing antibody in the MRL/lpr lupus model as the miRNA was not conserved in related species (**Figure 5A**). Compared with normal C57 mice, increasing proteinuria was observed in the anti-CXCL2Ab and untreated groups. However, the proteinuria was significantly reduced in the anti-CXCL2Ab group compared with the untreated group at 11 wk (*P* = 0.0044) (**Figure 5B**). H&E staining (**Figure 5C**) was used to assess the pathological changes in the kidney at the end of the study period. The major features analyzed included inflammatory cell infiltration, membrane proliferation, and arterial wall destruction (**Figures 5D–F**), which were all observed in the untreated group. By contrast, the occurrence of renal lesions was markedly mitigated in the anti-CXCL2Ab group. Therefore, anti-CXCL2Ab might alleviate kidney damage.

ANA is an important element in SLE patient categorization (16). Autoantibody levels are also related to lupus nephritis activity (17). Serum anti-dsDNA antibody and ANA levels were determined. The anti-dsDNA antibody and ANA levels were significantly lower in the plasma of anti-CXCL2Ab MRL/lpr mice than those in the untreated MRL/lpr mice (**Figures 5G, H**; *P* < 0.001). Hence, anti-CXCL2Ab treatment decreases serum immunological markers of SLE.

**Figure 6:**
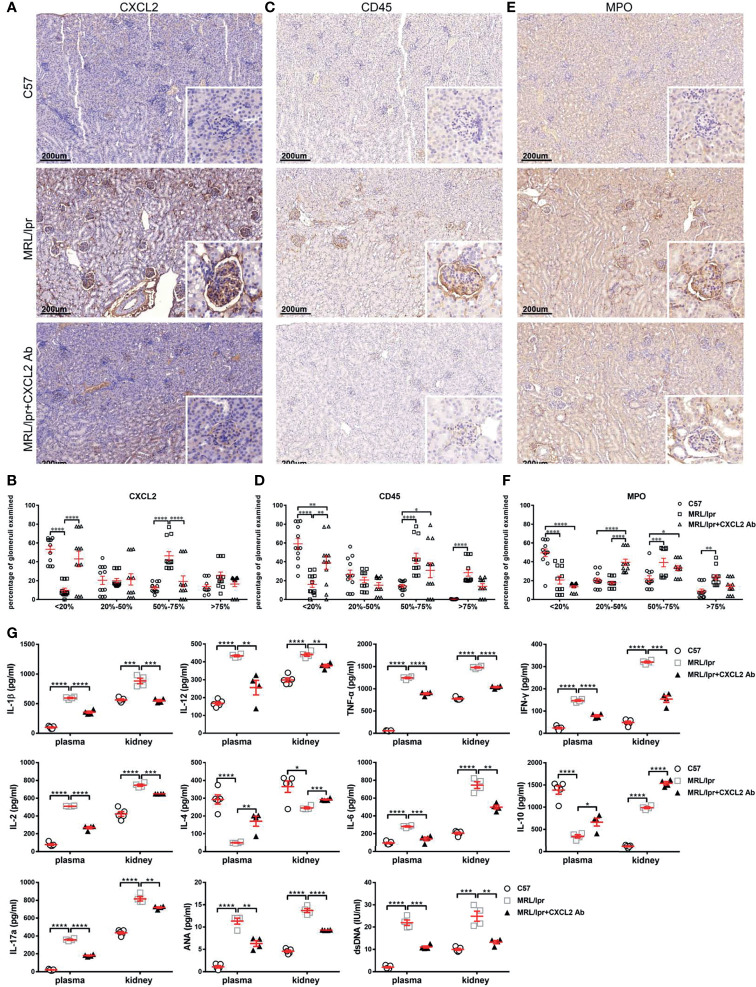
Treatment with CXCL2 antibody inhibits inflammatory cytokine production in the MRL/lpr lupus model. **(A, C, E)** Representative images of CXCL2 **(A)**, CD45 **(C)**, and MPO **(E)** staining of kidney sections of mice from different groups. Inserts, details of a typical staining of the glomerulus. **(B, D, F)** Quantitative analysis of CXCL2 **(B)**, CD45 **(D)**, and MPO **(F)** deposition in the glomeruli in kidney sections. The glomeruli were classified into four categories according to the degree of straining. The glomerulus ratios in each category in different groups are summarized (n = 4 animals/group, 3 sections/animal). Data are presented as the mean ± SEM. ****P < 0.0001; ***P < 0.001; **P < 0.01; *P < 0.05. **(G)** Concentrations of Th1 (including IL-1β, IL-2, IL-12, TNF-α, and IFN-γ), Th2 (including IL-4, IL-6, and IL-10) and Th17 (IL-17) cytokines in the plasma and renal tissue homogenates of mice from different groups, determined by ELISA (n = 4 animals/group). Data are shown as the mean ± SEM. ****P < 0.0001; ***P < 0.001; *P < 0.01; *P < 0.05.

Next, immunohistochemistry was used to determine the deposition levels of CXCL2, CD45, and MPO in glomerular sections (**Figures 6A–F**). As we expected, the glomerular CXCL2, CD45, and MPO levels of MRL/lpr mice were significantly higher than those of normal C57 mice (**Figures 6A–E**). The staining levels of CXCL2, CD45 and MPO in the glomeruli of MRL/lpr mice were significantly reduced by CXCL2Ab treatment (**Figures 6B–F**). Specifically, compared with untreated MRL/lpr mice, the percentage of III-degree glomeruli in MRL/lpr mice was significantly decreased, while the percentage of I degree glomeruli was significantly increased by CXCL2Ab, which were confirmed by the quantitative results of CXCL2 and CD45 (**Figures 6A–D**). In the quantitative results of MPO, the percentage of II-degree glomeruli was significantly increased by CXCL2Ab (**Figures 6E, F**). This confirmed that the glomerular damage of MRL/lpr mice was significantly reduced by CXCL2Ab, which was dependent on the decreased monocytes/neutrophils infiltration and activation.

The results of ELISA assay showed that the levels of IL-1β, IL-2, IL-6, IL-12, TNF-α, IFN-γ and IL-17 in the kidney and plasma were significantly down-regulated by CXCL2Ab, while IL-4 and IL-10 levels were significantly increased (**Figure 6G**). Therefore, CXCL2Ab was confirmed to regulate the secretion of inflammatory cytokines. These results indicate that CXCL2Ab is beneficial to relieve the immune disorder of SLE.

## Discussion

In this study, the abnormal expression of miRNAs in PBMCs of Tibetan population and its molecular mechanism involved in the progress of SLE were concerned. Differentially expressed miRNAs and mRNAs in PBMCs of SLE patients were detected by transcriptome sequencing. We detected significantly different miRNAs in Tibetan patients with SLE, including: miR-3942-3p, miR-4482-3p, miR-3165, miR-3164-3p, miR-4512, miR-4434, miR-99a-3p, and miR-4728-3p. Although miRNAs with abnormal expression of SLE have been reported, different studies have not found the same or highly consistent results. In our results, miR-3942-5p was reported as a good candidate diagnostic biomarker for class IV lupus nephritis (LN) (18). miR-3165 was found to be abnormally expressed in the serum of patients with grade IV lupus nephritis (19). The other 7 miRNAs in SLE have not been reported. In our study, miR-4512 was focused on. The results of sample validation and miRNA-mRNA analysis showed that miRNA-4512 was the most critical factor in the abnormal expression of PBMCs transcriptome in SLE patients. Furthermore, miRNA-4512 may be involved in disease progression of SLE by regulating TLR4 and CXCL2.

*In vitro* mechanism studies revealed that down-regulation of miR-4512 in monocytes and macrophages may promote inflammation and neutrophils NETosis by upregulating CXCL2 expression. Recent studies have shown that NETs formation is a major participant in the pathogenesis of systemic autoimmune diseases (6, 20). This is consistent with the increased expression of NETs-related proteins that we have observed in both Han and Tibetan SLE patients. The formation of NETs is accompanied by the exposure of a large number of autoantigens, which in turn induce or aggravate autoimmune diseases (21). In fact, NETs-related markers such as cfDNA were significantly upregulated in blood samples of SLE patients (22). Our research has also confirmed that the levels of serum-related indicators (MPO, cfDNA and NE) in SLE patients are abnormally up-regulated. Furthermore, the NETs induced by miR-4512 down-regulation was dependent on CXCL2.

The abnormal expression of chemokines is a major part of the immune disorders of SLE. For instance, CXCL10 and infiltrating CXCR3-positive cells have been implicated in various SLE manifestations (23). CCL21 and IP-10 are blood biomarkers of pulmonary involvement in SLE patients (24). CXCL13 was considerably upregulated in the kidney tissues of LN patients, which might be a therapeutic target in glomerulonephritis and SLE nephritis (25). The CXCR4/CXCL12 axis is a potential therapeutic target for SLE patients undergoing kidney and/or central nervous system pathogenesis (26). These studies suggest that abnormal levels of chemokine ligands are closely related to abnormal levels of inflammation and tissue damage in SLE. However, the mechanism of chemokine ligands in the formation of NETs in SLE patients remains unclear. In the study of chemokine-mediated NETs formation, CXCL1 was reported to rescue alcohol-induced immune disorders in polymicrobial sepsis by promoting the formation of NETs (27). In this study, we found that the NETs induced by miR-4512 down-regulation was dependent on the regulation of TLR4-CXCL2 signaling. It might be a new loop. Specifically, down-regulation of miR-4512 promotes up-regulation of CXCL2 and TLR4, and the CXCL2-TLR4 molecular axis further activates NF-κB-mediated chemokine and inflammatory cytokine expression, including CXCL2, which in turn promotes neutrophils recruitment to inflammatory sites and formation of NETs. The formation of loop may be a special pattern in chemokine-regulated NETosis. The formation of NETs is involved in regulating the recruitment of lung neutrophils to airway tissue, which depends on the TLR4/NF-κB pathway to stimulate airway cells to express CXCL1, CXCL2 and CXCL8 (28). The down-regulated expression of miR-4512 leads to the abnormal expression of CXCL2, which is likely to form a unique loop in the same way. This has been confirmed in studies of other chemokines. For example, the secretion of CXCL10 by CD4^+^, CD8^+^, natural killer (NK) and NK-T cells is dependent on IFN-γ, which is itself mediated by the IL-12 cytokine family (29). Under the influence of IFN-γ, CXCL10 is secreted by several cell types including endothelial cells, fibroblasts, keratinocytes, thyrocytes, preadipocytes, etc. Determination of high level of CXCL10 in peripheral fluids is therefore a marker of host immune response, especially T helper (Th)1 orientated T-cells (30). In tissues, recruited Th1 lymphocytes may be responsible for enhanced IFN-γ and tumor necrosis factor-α production, which in turn stimulates CXCL10 secretion from a variety of cells, therefore creating an amplification feedback loop, and perpetuating the autoimmune process (23). This suggests that CXCL2 may form an independent feedback loop in a similar manner.

Based on the immune regulation function of chemokines (including CXCL2) and its feedback loop mechanism, chemokines may be used as targets for the treatment of diseases related to immune disorders. In fact, blocking the interaction of chemokines and chemokine receptors has been proven to be beneficial for the treatment of autoimmune diseases. For example, the lymph node cells (LNC) of MRL/lpr mice was attenuated by CXCL13 blockade (25). Double blockade of CXCL12 and CCL2 shows the same efficacy as high-dose cyclophosphamide administration in murine proliferative LN (31). In this study, since miR-4512 was only expressed in human, and their target site in the 3’UTR of TLR4 and CXCL2 was not conserved in human and mouse, it would be difficult to directly testify the function of miR-4512 in the pathogenesis of SLE. Indeed, it was necessary to inhibit both TLR4 and CXCL2 in the SLE murine model to verify the function of miR-4512 in SLE indirectly. Although the role of TLR4 in SLE pathogenesis was well-defined in C57BL/6(lpr/lpr)-TLR4-deficient SLE model (32), TLR4 antibodies in a phase II clinical trial did not improve disease parameters of rheumatoid arthritis (33). These studies may suggest that antibody or antagonist was not sufficient to block TLR4 signaling as TLR4 are located both on cell surface and endosomal or lysosomal compartments. Moreover, evidences shown that the CXCL2 could be induced by LPS through TLR4 dependent pathway (34, 35), and the CXCL2 alone could induce NETs formation dependent on MAPK signaling pathway (36). Therefore, the CXCL2 would be a superior target in blocking TLR4-mediated inflammatory pathways when neutrophil recruitment and activation was concerned, especially when TLR4 was difficult to be inhibited by neutralizing antibody or small-molecule inhibitor. We thus only evaluate the effect of miR-4512 on MRL/lpr mice by blocking CXCL2 with neutralizing antibodies. Our results showed that proteinuria, ANA and anti-dsDNA antibody levels, pro-inflammatory cytokine secretion, and renal immune complex deposition in MRL/lpr mice were reduced by intraperitoneal administration of anti-CXCL2 neutralizing antibodies. In addition, CXCL2 blockade inhibited the production of MPO and CD45 in neutrophils of MRL/lpr mice. These results confirm that CXCL2 is a potent potential target in the treatment of SLE. It is also worth noting that the development of novel therapies or drugs to promote miR-4512 expression is also a novel strategy for SLE targeting therapy. In subsequent studies, the molecular mechanism of the abnormal expression of DEmiRNA in SLE patients will also provide new targets and insights for the treatment of SLE.

## Conclusions

Here, we detected the differentially expressed miRNAs in the PBMCs of Chinese Tibetan SLE patients. Among them, the abnormal down-regulation of miR-4512 in monocytes and macrophages was confirmed to promote the formation of SLE neutrophil extracellular traps by targeting CXCL2 and TLR4. Moreover, CXCL2 blockade was confirmed to reduce kidney damage in MRL/lpr mice. These data are the first to characterize the mechanism of miRNA in Tibetan SLE patients and provide new insights into the general pathogenesis of SLE patients.

## Data Availability Statement

Sequencing data sets have been deposited in the gene expression omnibus (GEO) data repository under the accession numbers GSE175839, GSE175840, GSE175841.

## Ethics Statement

The study design was approved by the Ethics Committee of the Second Affiliated Hospital of Kunming Medical University. The patients/participants provided their written informed consent to participate in this study. The experiment was conducted in a laboratory animal facility of Kunming Medical University and approved by the Animal Research Committee of Kunming Medical University (No. kmmu2021724).

## Author Contributions

BY was involved in the majority of experiments, data statistics and analysis, and drafting and revision of the manuscript. XH was involved in experiment supervision and management, data analysis, and revision of the manuscript. SX, LL, WW, and YD performed bioinformatics analysis of the RNA sequencing data and differential expression analysis. MG, LY, WC, MY, and YW were involved in the acquisition of human samples, data capture, and analysis of clinical data. DD was involved in the conception and design of the study and interpretation of data. All authors critically revised the manuscript and approved the final version of the manuscript.

## Funding

This research was supported by National Natural Science Foundation of China (grant no. 81860552, 31860256), Applied Basic Research Programs of Yunnan Province (grant no. 2018FB129).

## Conflict of Interest

The authors declare that the research was conducted in the absence of any commercial or financial relationships that could be construed as a potential conflict of interest.

## Publisher’s Note

All claims expressed in this article are solely those of the authors and do not necessarily represent those of their affiliated organizations, or those of the publisher, the editors and the reviewers. Any product that may be evaluated in this article, or claim that may be made by its manufacturer, is not guaranteed or endorsed by the publisher.

## Supplementary Material

The Supplementary Material for this article can be found online at: https://www.frontiersin.org/articles/10.3389/fimmu.2021.756825/full#supplementary-material
