# Supplementary figures and images for: Decreased miR-4512 Levels in Monocytes and Macrophages of Individuals With Systemic Lupus Erythematosus Contribute to Innate Immune Activation and Neutrsophil NETosis by Targeting TLR4 and CXCL2
Source: Front Immunol. 2021 Oct 14;12:756825. doi: 10.3389/fimmu.2021.756825 (PMC8552026; doi:10.3389/fimmu.2021.756825)

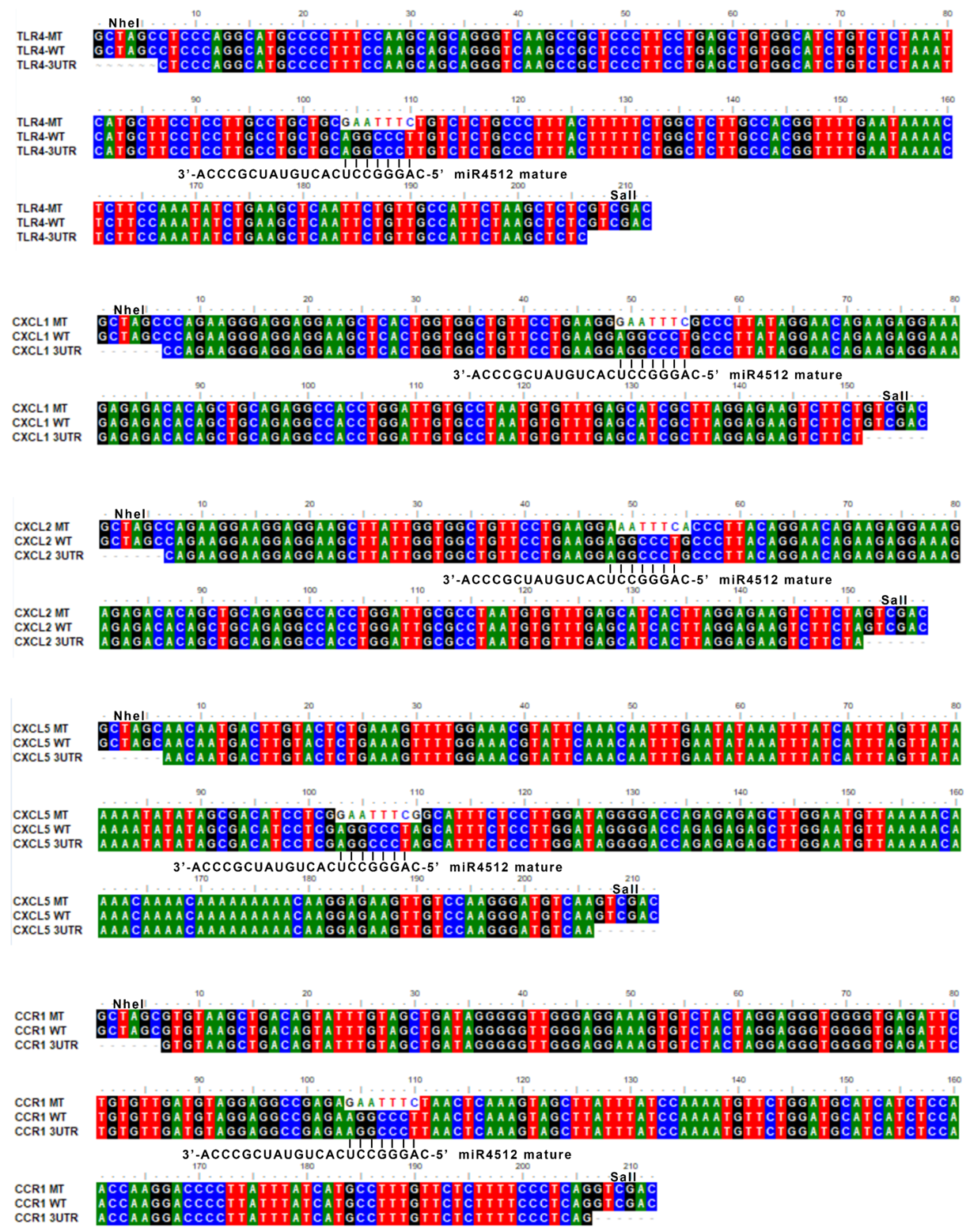

Supplement: Supplementary file 1 [file Image_1.png]

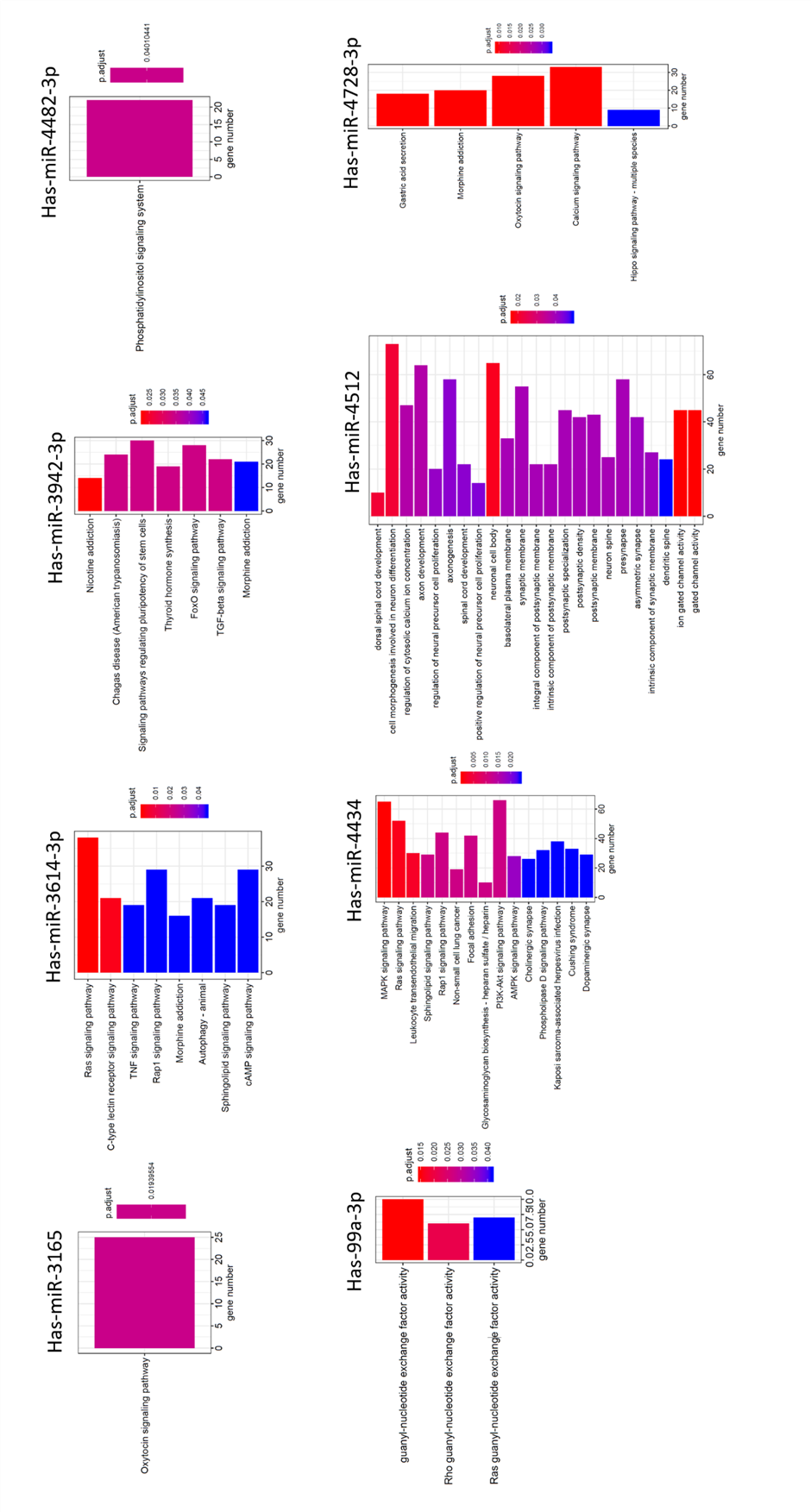

Supplement: Supplementary file 2 [file Image_2.png]

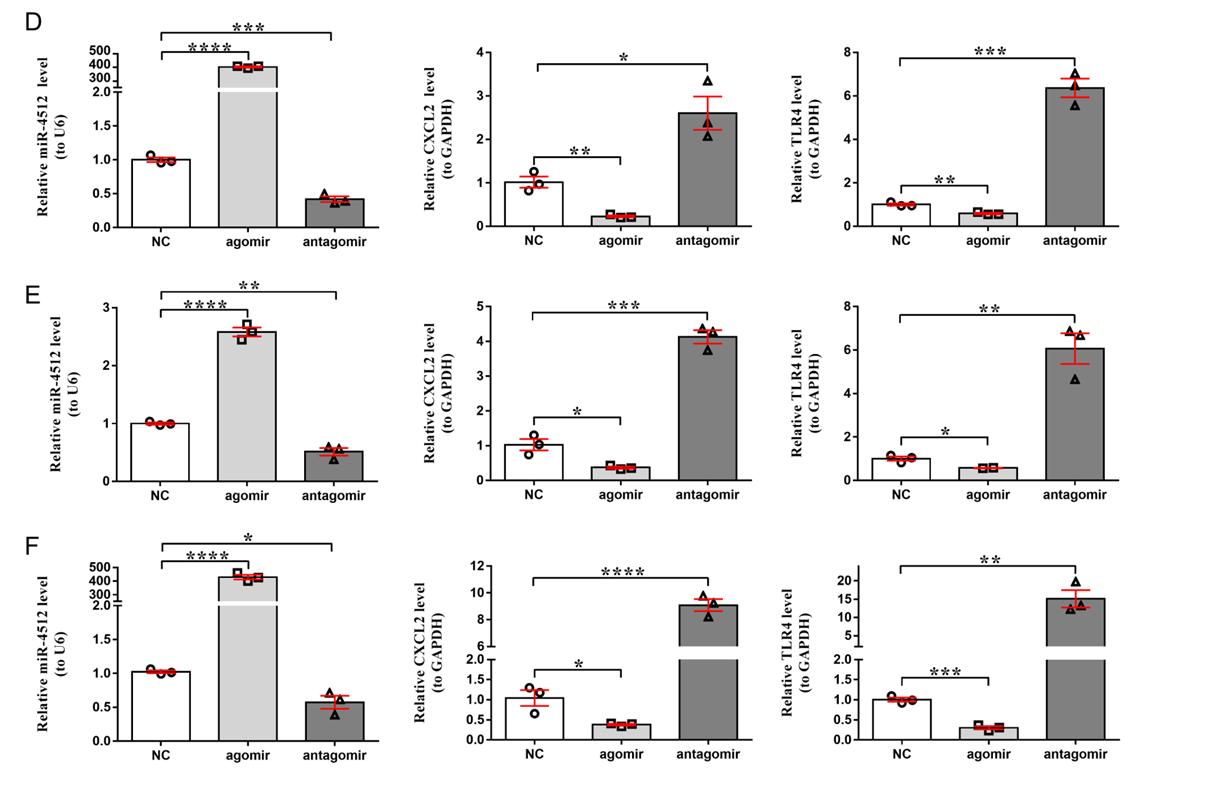

Supplement: Supplementary file 3 [file Image_3.png]

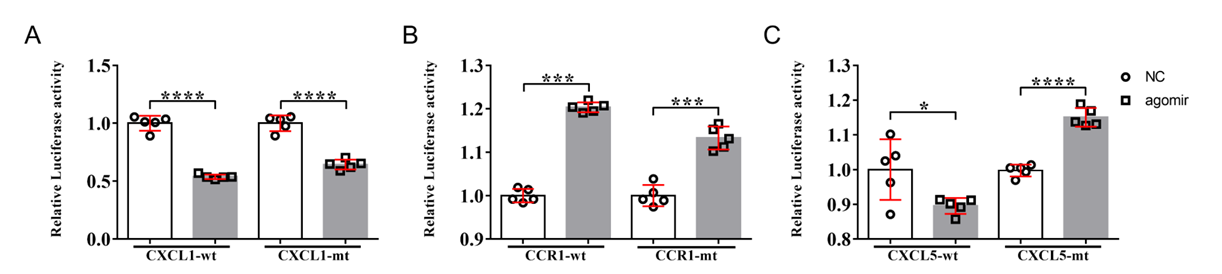

Supplement: Supplementary file 4 [file Image_4.png]
