# Supplementary material for: Decreased miR-4512 Levels in Monocytes and Macrophages of Individuals With Systemic Lupus Erythematosus Contribute to Innate Immune Activation and Neutrsophil NETosis by Targeting TLR4 and CXCL2
Source: Front Immunol. 2021 Oct 14;12:756825. doi: 10.3389/fimmu.2021.756825 (PMC8552026; doi:10.3389/fimmu.2021.756825)
Supplement: Supplementary file 5 [file Presentation_1.pdf]

Western blot: 250ul RIPA lysis buffer was added into the cells to be detected of each group, and then cells were put onto the ice to be fully split. Then they were centrifugated at 16000g for 10min and the supernatant was removed. The concentration of the obtained protein was detected with BCA protein quantitative kit ((P0010, Beyotime, China). 12% separating gel and 5% stacking gel was prepared according to the instruction of gel-prepared kit for the conduction of SDS-PAGE electrophoresis (SDS-PAGE loading buffer (5X), P0015L, Beyotime, China). 25ug of total protein was put into each hole and the electrophoresis (80V, 40min; 100V, 100min) was conducted. After the electrophoresis, the protein was electroporated (200mA, 90min)onto 0.2um PVDF membrane (IPVH00010, EMD Millipore, Billerica, MA, USA). Then the membrane was drawn out, and was blocked under the room temperature for 2h using 5% skimmed milk and 0.1% Tween-20 TBS（TDST). The membrane was washed by TBST for 6 min in triplicate. The primary antibody TLR4（1:1000, AF7017, Affinity, Jiangsu, China), NE(Neutrophil Elastase (E9C9L) XP® Rabbit mAb，89241S，CST，USA), β-Actin(1:5000,ab6276, Abcam) were diluted with TBST containing 5% BSA and then they were incubated by shaking under 4℃ overnight. Then the primary antibody was removed and TBST was used to rinse for 6 min in triplicate. Then the second antibody (1:5000 dilution, Sigma, MA, USA) marked with Horseradish Peroxidase was diluted by TBST. Containing 5% BSA. Then it was incubated under room temperature for 2h. The second antibody was removed and it was washed by TBST for 15min in triplicate. It was put into visualization processing with electrochemiluminescence detection reagent (Beyotime biotechnology company), and then it was exposed and photographed with Gel Imaging System (QuickGel 6100, Monad, Wuhan, China). Finally, the software Image J1.43 was used to measure the average gray value. And the relative expression of protein was calculated by the ratio of average gray value and β-Actin gray value.
