# Supplementary material for: Decreased miR-4512 Levels in Monocytes and Macrophages of Individuals With Systemic Lupus Erythematosus Contribute to Innate Immune Activation and Neutrsophil NETosis by Targeting TLR4 and CXCL2
Source: Front Immunol. 2021 Oct 14;12:756825. doi: 10.3389/fimmu.2021.756825 (PMC8552026; doi:10.3389/fimmu.2021.756825)
Supplement: Supplementary file 6 [file Table_1.docx]

Table 1. General clinical data of subjects (x̅±s)

| Group | Tibetan patients with SLE Group | Tibetan patients with healthy Group | Han patients with SLE Group | Han patients with healthy Group | p-value |
| --- | --- | --- | --- | --- | --- |
| Number | n=10 | n=9 | n=9 | n=8 |  |
| Age | 46.1±16.42 | 39.70 ± 3.757 | 35±13.67 | 29.78 ± 1.372 | 0.3369 |
| Female/Male | 9/1 | 10/0 | 8/1 | 9/0 | 1.0000 |
| Disease duration | 7.8±2.62 | - | 8±5.43 | - | 0.9184 |
| Altitude | 3171.6±222.64 | 3079 ± 74.35 | 1627±124.94 | 1631 ± 47.46 | < 0.0001 |
| ANA, n (%) | 9(90%) | - | 9(100%) | - | 1.0000 |
| Anti-ds-DNA, n(%) | 3(30%) | - | 4(44.44%) | - | 0.6499 |
| Anti-Sm, n (%) | 3(30%) | - | 3(33.33%) | - | 1.0000 |
| Anti-Ro, n (%) | 4(40%) | - | 3((33.33%)) | - | 1.0000 |
| Anti-SSA, n (%) | 3(30%) | - | 2(22.22%) | - | 1.0000 |
| Anti-U1RNP, n (%) | 4(40%) | - | 4(44.44%) | - | 1.0000 |
| Corticosteroid therapy, n (%) | 10(100%) | - | 9(100%) | - | 1.0000 |
| Immunosuppressive therapy n (%) | 6(60%) | - | 7(77.77%) | - | 0.6285 |
| Disease activity (SLEDAI,  mean ± SD) | 7.400±3.03 | - | 9.111±4.73 | - | 0.3558 |
