# Supplementary material for: Decreased miR-4512 Levels in Monocytes and Macrophages of Individuals With Systemic Lupus Erythematosus Contribute to Innate Immune Activation and Neutrsophil NETosis by Targeting TLR4 and CXCL2
Source: Front Immunol. 2021 Oct 14;12:756825. doi: 10.3389/fimmu.2021.756825 (PMC8552026; doi:10.3389/fimmu.2021.756825)
Supplement: Supplementary file 7 [file Table_2.docx]

Table 2. Primer sequences in RT-qPCR

| IFN-γ F | GTTTTGGGTTCTCTTGGCTGTTA | CCR1 F | CAGAAGGTGAACGAGAGGGC |
| --- | --- | --- | --- |
| IFN-γ R | TCATCTCGTTTCTTTTTGTTGCT | CCR1 R | CCAAGGAGTACAGAGGGGGC |
| TNF-α F | CTGCACTTTGGAGTGATCGG | CXCL1 F | CTGCTCTCTCCGCCGCCCC |
| TNF-α R | ACAACATGGGCTACAGGCTT | CXCL1 R | GCTTTCCGCCCATTCTTGA |
| IL-1 F | CTGCTGAAGGAGATGCCTGAGA | CXCL2 F | CAGAAGGTGAACGAGAGGGC |
| IL-1 R | GCAATAAACAAGTTTGGATGGG | CXCL2 R | CCAAGGAGTACAGAGGGGGC |
| IL-2 F | TGCTGGATTTACAGATGATTTTGA | CXCL5 F | AGCTGCGTTGCGTTTGTTT |
| IL-2 R | TGATATTGCTGATTAAGTCCCTGG | CXCL5 R | TTCCTTGTTTCCACCGTCC |
| IL-4 F | CTGCTTCCCCCTCTGTTCTTC | TLR4 F1 | AGGTTTCCATAAAAGCCGAAAG |
| IL-4 R | GTGATATCGCACTTGTGTCCG | TLR4 R1 | CAATGAAGATGATACCAGCACG |
| IL-6 F | TTCGGTCCAGTTGCCTTCTC | TLR4 R2 | TTCAGGGACAGGTCTAAAGAGA |
| IL-6 R | GTGCCTCTTTGCTGCTTTCA | TLR4 F2 | TCCAGCAACAAGATTCAAAGTA |
| IL-10 F | ACGGCGCTGTCATCGATTT | miR-4512 | CAGGGCCTCACTGTATCGCCCA |
| IL-10 R | TCCACGGCCTTGCTCTTGT |  |  |
| IL-12 F | TGGAATTAACCAAGAATGAGAG |  |  |
| IL-12 R | AAGCATGAAGAAGTATGCAGAG |  |  |
| IL-17 F | TACAACCGATCCACCTCACCTT |  |  |
| IL-17 R | GACACCAGTATCTTCTCCAGCC |  |  |
